# Influence of particle size and dielectric environment on radiative lifetimes of colloidal cadmium selenide single photon emitters

**DOI:** 10.1038/s41598-025-99148-9

**Published:** 2025-04-26

**Authors:** V. Manojkumar, Geetha K. Varier, Radhika Vathsan, P. Nandakumar

**Affiliations:** Department of Physics, Birla Institute of Technology and Science, Pilani, K K Birla Goa Campus, Zuarinagar, Sancoale, Goa 403726 India

**Keywords:** Colloidal quantum dots, Single photons, Second-order correlation function, Spontaneous emission rate, Correlation coefficients, Single photons and quantum effects, Quantum optics

## Abstract

High repetition rate single-photon emitters are essential for all-optical quantum information processing, communications and metrology. The spontaneous emission lifetimes of colloidal cadmium selenide quantum dots are typically of the order of 10 ns, severely limiting their brightness and therefore their potential applications in quantum devices. Here we report on single-photon emission at room temperature with nanosecond lifetime from cadmium selenide quantum dots embedded in a polymer matrix. The study shows that the emission lifetime can be tuned by appropriately choosing the particle size and the dielectric constant of the surrounding medium. The quantum dots are synthesized using a green synthesis protocol and surface passivated using oleic acid. A Hanbury Brown and Twiss setup attached to an in-house constructed confocal microscope is used to efficiently couple and characterize the single-photon emission from an array of quantum dots. Detailed analysis of the second-order correlation function ($g^{(2)}(\tau )$) of single-photon emission from cadmium selenide quantum dots reveals the particle-size dependence of emission lifetimes. The study also shows that the quality of single-photon emission, as revealed by $g^{(2)}(0)$, reduces with increasing particle-size in the strongly confined regime.

## Introduction

Physical^1–3^ and chemical^4,5^ properties of quantum dots (QDs) undergo significant changes due to the confinement of charge carriers within the material^6^. The confinement effect is pronounced when the size of the quantum dots is less than the de Broglie wavelength of the charge carriers, making an increase in the exciton binding energies^7^. Quantum confinement results in remarkable changes in the density of states, leading to the formation of discrete energy levels rather than the continuous energy bands normally observed in bulk solids^8^. Semiconductor QDs, with their discrete energy levels, are one of the potential candidate materials for developing single-photon sources for quantum technologies. On-demand single-photon sources are integral to any optical implementation of quantum technologies, crucial in quantum communication^9^ and quantum metrology^10^, and are part of any quantum optics lab focusing on experiments on foundations of quantum physics^11^.

The quest for developing on-demand single-photon sources with high brightness (high repetition rate of single photons) has gained much momentum in recent years^12,13^. A higher repetition rate of photons is normally achieved by increasing the spontaneous emission rate by coupling single-photon sources to plasmonic cavities^14^. However, this technique inherently leads to a higher probability of multiple photons emission^15^. Epitaxially grown cadmium selenide quantum dots (CdSe QDs) on zinc selenide have suppressed non-radiative phenomena at higher temperatures, thereby decreasing the radiative lifetime^16^. However, epitaxial QDs are mostly known for emitting single photons at cryogenic temperatures and their scalability is limited. Also, molecular beam epitaxy is a complicated and expensive technique. Here, we report on the nanosecond spontaneous emission lifetimes observed in colloidal CdSe QDs immersed in a polymer matrix. To our knowledge, single-photon emission from colloidal CdSe QDs at room temperature with size-dependent lifetimes has not been reported so far.

Cadmium selenide is a group II-VI chalcogenide compound with a direct bulk bandgap of 1.74 eV, which can be increased by decreasing the crystal size. In CdSe QDs, each selenium anion is surrounded by four cadmium cations and vice versa to form a tetrahedral structure that resembles $sp^3$ hybridization. This indicates the presence of characteristics associated with both covalent and ionic bonding^17^. Colloidal synthesis of CdSe QDs is usually done by the conventional hot-injection method, which involves the rapid mixing of reagents at high temperatures^18^. However, the difficulty in introducing large amounts of reagents into the mother solution in a very short time creates an insurmountable drawback in this type of synthesis. In addition, the reaction temperature needs to decrease after the injection step to confine nucleation to a brief interval and slow down subsequent growth of nanocrystals. Since the cooling rate does not scale linearly with the reaction volume, the uniformity in the QD size is affected^19^. A two-fold change in the size of QDs typically results in a shift of tens of nanometers in emission wavelengths, which poses a severe limitation in applications where photon indistinguishability is crucial^20^. Some of these drawbacks can be overcome by using heat-up synthesis techniques. This involves mixing two precursors at higher temperatures and promotes slow growth of nanocrystals, for better control over the size.

**Fig. 1:**
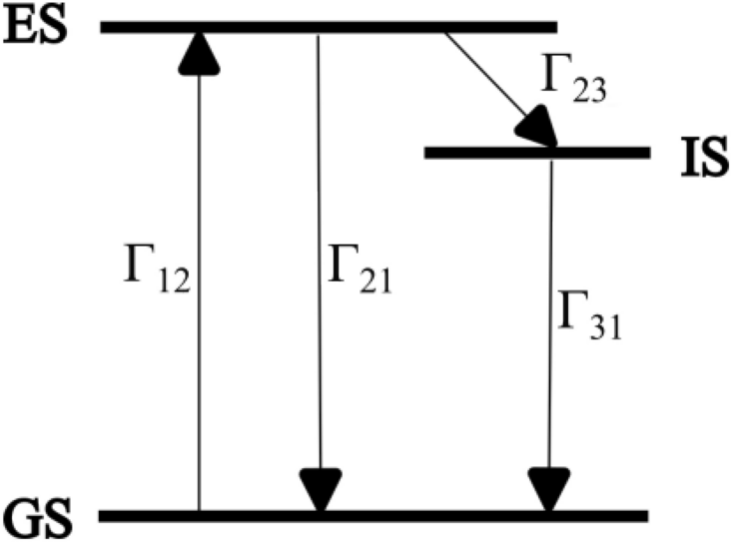
Energy-level diagram for a three-level system of colloidal CdSe QDs. An electron is excited from the ground state (GS) to an excited state (ES) with the transition rate $\Gamma _{12}$ upon absorbing a photon of energy equivalent to (ES - GS). This electron either transits to GS with the rate $\Gamma _{21}$ by emitting a photon or non-radiatively transits to an intermediate state (IS) with the rate $\Gamma _{23}$. Further relaxation to the GS with rate $\Gamma _{31}$ occurs radiatively or non-radiatively.

Colloidal CdSe QDs are associated with lattice defects that lead to various nonradiative pathways, affecting the efficiency of fluorescence emission. This phenomenon is depicted with the presence of an intermediate energy level (IS) between the ground state (GS) and the excited state (ES), in Fig. 1^21^. The probability of electronic transition mediated by the IS increases the quenching of emission. This quenching can be reduced by passivating the surface of the QDs with appropriated surfactants. Passivation reduces the likelihood of non-radiative recombination processes occurring due to the presence of surface defects or dangling bonds, that might trap charge carriers^22^. Surfactants such as oleic acid contain a carboxylate chain and have an anionic nature^23^; they attach to the excess cadmium cations on the surface to prevent the aggregation of QDs as well. Oleic acid is proven to be effective in preventing agglomeration compared to other passivating agents^24^. Fluorescence intermittency, or “blinking” associated with nonradiative transitions, is a major drawback of colloidal quantum dot-based single photon sources. Different mechanisms contribute to blinking in semiconductor quantum dots. One of the commonly observed phenomena in charged quantum dots is Auger recombination, where the energy from electron-hole pair recombination is transferred to another electron (or hole), which is subsequently excited to a higher energy level. Blinking is also attributed to band edge carrier trapping (BC-blinking) and hot electron trapping (HC blinking)^25–27^. All these different processes can coexist with one another. Recent works have demonstrated the control of blinking and techniques to reduce and eliminate blinking^28–30^. The techniques include the use of passivating agents to eliminate BC blinking^28^ and the use of a sufficiently thick inorganic shell layer to eliminate Auger blinking^29^. Optical techniques such as exposure to short-duration infrared radiation are found to reduce blinking^30^.

The present work focuses on increasing the repetition rate of single-photon emission from colloidal CdSe QDs without using an external cavity. So far, the studies on colloidal CdSe QDs emitting single photons show lifetime of the order of ten nanoseconds^31^. Previously, the lifetimes shorter than nanoseconds for core/shell quantum dots are obtained^32,33^, but they are limited by the photon repetition rate due to the larger contribution of slow-decay components of lifetimes. In this work, an order of magnitude reduction in the spontaneous emission lifetime is achieved by (i) making use of QDs in the intermediate confinement regime and (ii) immersing QDs in a medium of higher dielectric constant. This study is conducted by exciting the QDs with a continuous wave (CW) laser and does not require a high-power ultrafast laser. The correlation between the spontaneous emission lifetime and size of the individual CdSe QDs is elucidated in this study by comparing the distribution of the lifetime from QDs in an array with their size distribution. The suppression of photo-bleaching by using a surrounding medium provides an added advantage for the QDs to emit single photons at a constant rate for longer duration. Experiments are carried out using a confocal microscope attached to a Hanbury Brown and Twiss (HBT) interferometer. An array of well-separated QDs in the form of a thin film is studied using the setup which allows for probing different QDs by moving the focal spot. Further, we elucidate the correlation between the lifetime and size of the individual CdSe QDs by comparing the distribution of the lifetime of QDs in an array with their size distribution. Efficient and simultaneous coupling of an array of single-photon emitters to the detectors^34^ at room temperature is of paramount importance in quantum technologies.

## Experimental section

### Synthesis and sample preparation of colloidal CdSe QDs

Colloidal CdSe QDs are synthesized using a green synthesis method as follows^35^. Selenium precursor (1 ml, 5 mM) is prepared by dissolving elemental selenium (> 99.5 % purity, Sigma Aldrich) in olive oil at $200\,^\circ$C for 2 h and subsequently cooling to room temperature. Cadmium precursor (5 ml, 10 mM) is prepared by dissolving cadmium oxide powder (> 99% purity, Nice Chemicals) in olive oil at $300\,^\circ$C for 30 min. Oleic acid (60 $\upmu$l) (> 97 % purity, Spectrochem) is added to the cadmium precursor while heating. Cooled selenium precursor is then immediately added to hot cadmium precursor, with a sudden lowering of temperature to $280\,^\circ$C. This solution is heated for 1 hour at $300\,^\circ$C. The hot solution is immediately poured into acetone (40 ml) (Sigma Aldrich), forming a precipitate. This is centrifuged at 2000 rpm for 10 minutes to allow the precipitate to separate from the supernatant. The supernatant is decanted, and this process is repeated four more times to remove the residual olive oil. The final precipitate is added to toluene (2 ml) (99.9 % purity, Sigma Aldrich) for characterization, without further purification. Absorption and photoluminescence spectra of the synthesized samples are presented in the Supplementary Information (Figures S2 (a) and S2 (b)).

The procedure to prepare a thin film of CdSe QDs for confocal microscope imaging is as follows:

Polymethyl methacrylate (PMMA) powder (50 mg) (molecular weight 120,000, Sigma Aldrich) is added to toluene (1 ml). This is heated at $50\,^\circ$C until a clear transparent solution is obtained. The polymer solution is added to concentrated CdSe QD solution (20 $\upmu$l) to prepare a stock solution. The stock is diluted in toluene (1 ml) and is sonicated for 40 minutes. This mixture (20 $\upmu$l) is spin-coated at 1000 rpm for 60 seconds on a coverslip to form a thin film of uniform thickness. The obtained transparent polymer film contains monodispersed CdSe QDs and is used for confocal microscope imaging. The dilution of CdSe QDs is so chosen that the spin-coated film has a particle density of less than 1 $\upmu$ $\hbox {m}^{-2}$ area.

### Confocal Hanbury Brown and Twiss microscope for characterization of single-photon emission

**Fig. 2:**
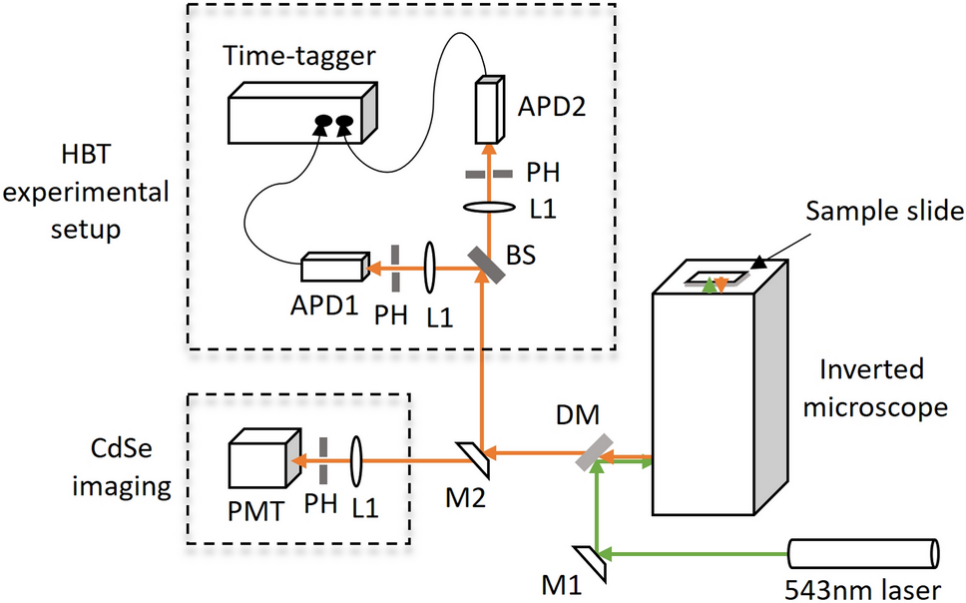
Experimental setup for imaging and detection of single photon emitters. M1—fixed mirror, M2—flip mirror; DM—dichroic mirror, BS—50% beam splitter, L1—lens, PH—pinhole, APD—avalanche photodiode, PMT—photomultiplier tube.

**Fig. 3:**
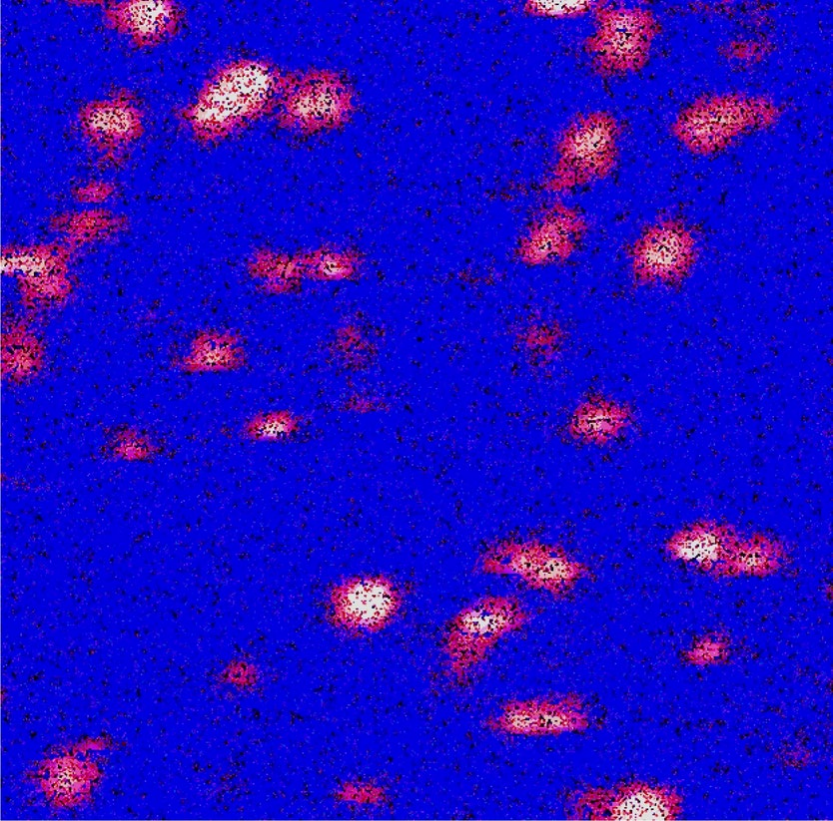
Confocal microscope image of colloidal CdSe QDs of 16 $\upmu\hbox {m}^2$ area. Each bright spot has a diameter of about 280 nm, implying diffraction-limited imaging.

The spin-coated CdSe QDs film is imaged using a confocal microscope that was constructed in-house^36^. A schematic diagram of the experimental setup is shown in Fig. 2. A continuous wave laser beam of wavelength 543 nm is used to excite the particles in the film. This beam is focused onto the film by a 60X oil immersion objective (NA = 1.25, UPlanFLN 60X, Olympus) attached to Olympus IX71 inverted microscope. ScanImage software (Vidrio Technologies) is used to communicate with the data acquisition card (NI-USB-6356) to control both the XY scanning galvo-mirror system (GVS002/M, Thorlabs) (not shown in the figure) and photomultiplier tube (PMT) (H7732-10, Hamamatsu Photonics). Fluorescence light emitted from the film is transmitted through a dichroic mirror (DM) (ZT488/543rpc, Chroma Technology Corp) and is allowed to fall on the PMT. A typical 2D image of CdSe QDs film as shown in Fig. 3 is obtained by raster scanning the sample using XY scanning galvo-mirrors system.

Once the confocal image of CdSe QDs is obtained, a particular QD is selected and the laser beam is continuously focused onto it through the microscope objective. The fluorescence from this QD is then directed into the HBT setup with the help of the flip mirror M2. The HBT setup consists of a 50–50 beam splitter (BS) from which two equal-intensity output beams are focussed onto the active area of avalanche photodiodes APD1 and APD2 (360 ps resolution, SPCM AQRH-14FC, Excelitas Technologies), through lenses L1 of 5 cm focal length. Pinholes PH placed after L1 in each arm eliminate the light from the out-of-focal-plane reaching APDs. These APDs generate an output TTL pulse upon each photon-detection event, the timings of which are recorded using a time-tagger (81 ps resolution, ID800, ID Quantique). An algorithm written in Matlab constructs a histogram of the number of photons reaching the detectors with time delay $\tau = |t - t'|$, where *t* and $t'$ are the recorded time of arrival of photons in APD1 and APD2 respectively. The quality of single-photon emission is studied by computing the normalized second-order correlation function $g^{(2)}(\tau )$. This relates the probability of detecting a photon at a time *t* to the probability of detecting another photon at a later time $t'$ and is defined by

1 $$\begin{aligned} g^{(2)}(\tau ) = \frac{\langle n_1(t) n_2(t+\tau ) \rangle }{\langle n_1(t) \rangle \langle n_2(t+\tau ) \rangle } \end{aligned}$$

where $\langle n_1 \rangle$ and $\langle n_2 \rangle$ are the time-averaged count rates of photons in APD1 and APD2 respectively. For all practical single-photon sources, $g^{(2)}(0)$ is close to zero, the expected value for an ideal single-photon source.

## Results and discussion

### Imaging of CdSe QDs

The spin-coated film of monodispersed CdSe QDs in PMMA polymer matrix is imaged using the confocal microscope setup. A sample image of the QDs from an area of 16 $\upmu \hbox {m}^2$ is shown in Fig. 3. The average diameter of the bright spots is 280 nm, which is the expected diffraction-limited spot size. The horizontal stripes seen on bright spots in the confocal image are due to the photoluminescence blinking^37^.

### Second-order correlation function

**Fig. 4:**
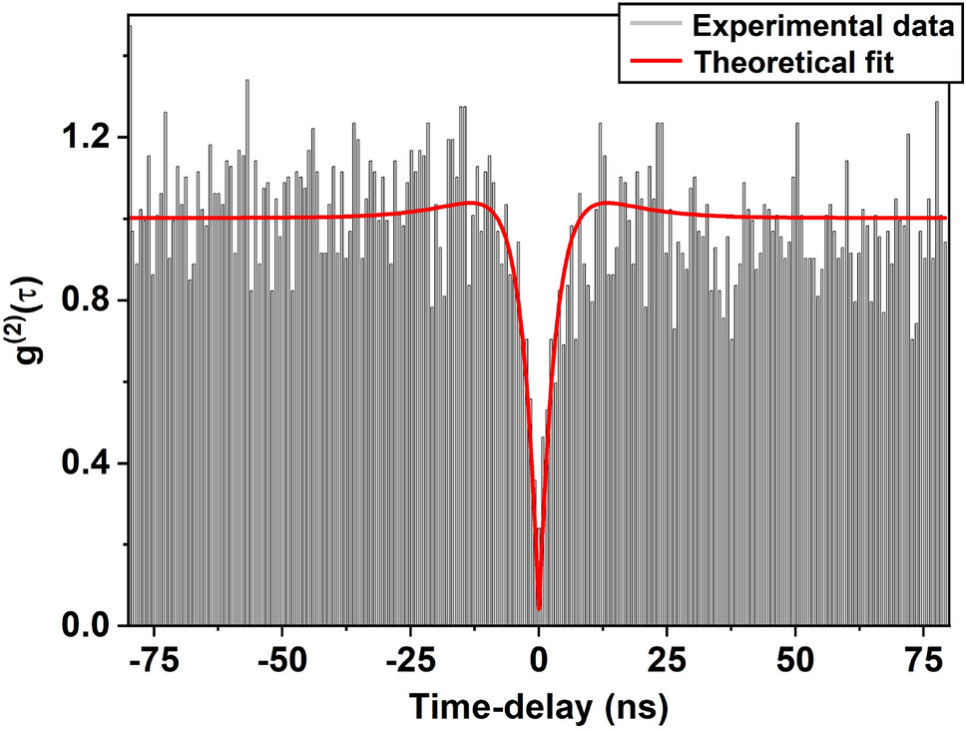
Normalized second-order correlation function $g^{(2)}(\tau )$ as a function of time-delay $\tau$ for a single colloidal CdSe QD immersed in PMMA matrix. Experimental data is fitted to Eq. (2) (red curve). The value of $g^{(2)}(0)$ less than 0.5 indicates the presence of a single quantum emitter inside the confocal volume.

Single-photon emission from individual CdSe QDs is characterized using the HBT setup (Fig. 2). A typical plot of the second-order correlation function $g^{(2)}(\tau )$ is shown in Fig. 4. Each column in the figure refers to the number of photon-pair detection events in APD1 and APD2 with a time delay $\tau = |t - t'|$. The second-order correlation function for a three-level system is related to the transition lifetimes by^21,38^

2 $$\begin{aligned} g^{(2)}(\tau )=1-(1+b)e^{-\lambda _1 |\tau |}+be^{-\lambda _2 |\tau |} \end{aligned}$$

where $\lambda _1$ is the decay constant corresponding to ES $\leftrightarrow$ GS transition. $\lambda _1 \sim \Gamma _{12} + \Gamma _{21}$ where $\Gamma _{12}$ and $\Gamma _{21}$ are the rate constants corresponding to absorption and subsequent spontaneous emission from level ES to level GS, respectively (see Fig. 1). However, for low power excitation, as is the case here, the decay constant $\lambda _1$ is dominated by the decay rate of the excited state; $\Gamma _{21}$ being an order magnitude higher than $\Gamma _{12}$^21^. $\lambda _2$ is the transition rate mediated by the ES $\rightarrow$ IS $\rightarrow$ GS transition. The second term of Eq. (2) reflects photon-antibunching and causes a dip to appear in the correlation function at $\tau = 0$. This behavior arises because the QD re-emits a photon only after the excited state has decayed by emitting the first photon. The third term in the above equation is responsible for the blinking of emission, which arises due to the inherent defect states of colloidal CdSe QDs^39^. The coefficients corresponding to the second and third terms represent the amplitude of transitions to ES and IS respectively.

The correlation data in Fig. 4 is fitted to the behavior predicted by Eq. (2) for a three-level system after deconvolution (red curve) with the instrument response function of 360 ps. This fit gives the parameters *b*, $\lambda _1$ and $\lambda _2$ of Eq. (2). The lifetime $t_1 = 1/\Gamma _{21}$ of the excited state, as discussed earlier, is approximately equal to $1/\lambda _1$. The above analysis is carried out on 64 individual CdSe QDs to determine the excited state lifetimes $t_1$. It is found that the single-photon emission lifetime $t_1$ in these particles vary from 0.4 ns to 5.2 ns at room temperature. These values agree well with the an average lifetime of 2 ns measured in these samples using time-resolved photoluminescence spectroscopy.

**Fig. 5:**
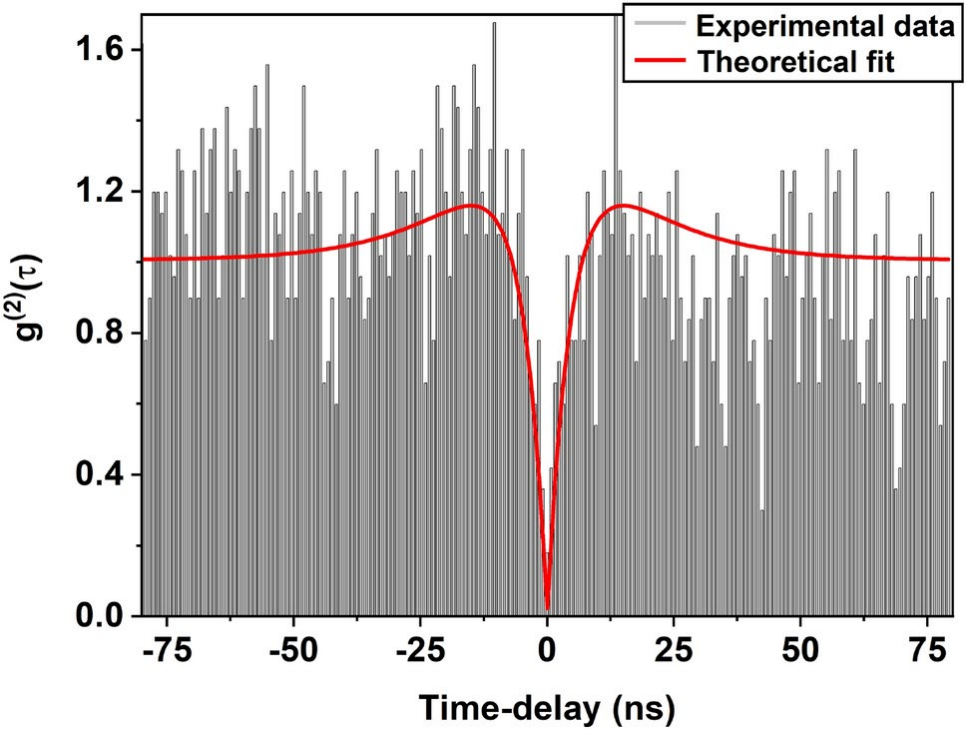
$g^{(2)}(\tau )$ as a function of time-delay $\tau$ for free standing colloidal CdSe QDs. The continuous red line is a fit to Eq. (2).

The value of $g^{(2)}(0)$ in Fig. 4 is close to zero. The non-zero value of $g^{(2)}(0)$ could arise due to various reasons, such as (i) a small probability of multiple photons generated from the biexciton states^40^, (ii) a fraction of scattered laser light reaching the detectors that increase with the incident power. The noise around $\tau = 0$ in $g^{(2)}(\tau )$ plot could arise from (i) the photons emitted at random times due to the slow decay component $t_2$^13^, (ii) from the shot noise associated with the laser and APDs. The appearance of wings around the dip is associated with the third term of Eq. (2) that signifies the exhibition of photoluminescence blinking.

We have also carried out single-photon emission studies from free standing colloidal CdSe QDs prepared by the same same procedure as in the previous case, but without the PMMA matrix for comparison. Figure 5 shows a typical second-order correlation function plotted for single-photon emission from a CdSe quantum dot, without PMMA matrix. As the free standing quantum dots are more prone to photo-bleaching, the signal-to-noise ratio in these data are lower. The value of $t_1$ determined from Eq. (2) to this data is $\approx$ 8 ns, which is much higher than those QDs immersed in PMMA medium.

### Particle-size and radiative lifetime

**Fig. 6:**
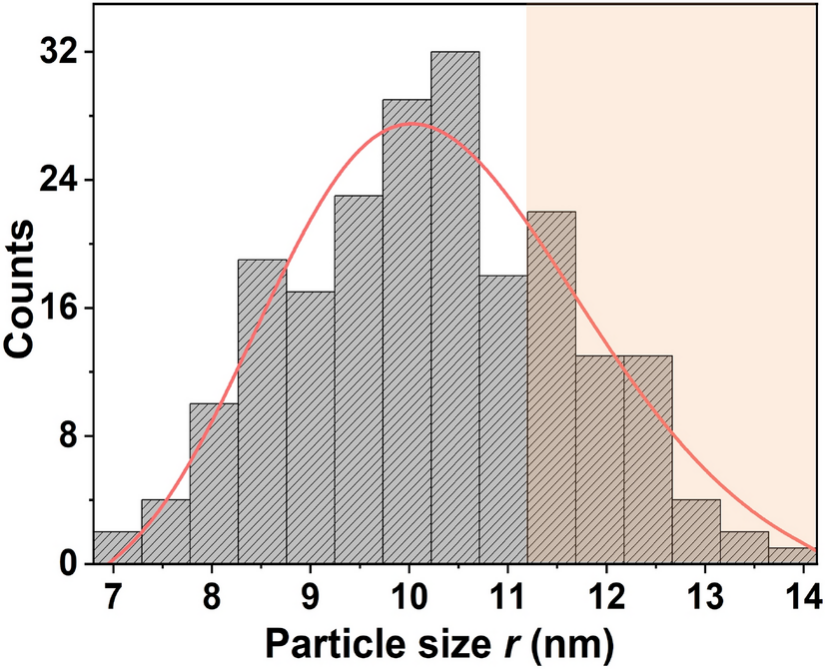
Size (diameter) distribution obtained from SEM image for colloidal CdSe QDs. This distribution is fitted using log-normal probability density function of Eq. (6), shown by the red curve. The color of the region above 11 nm is slightly changed to differentiate the confinement regime for CdSe QDs.

**Fig. 7:**
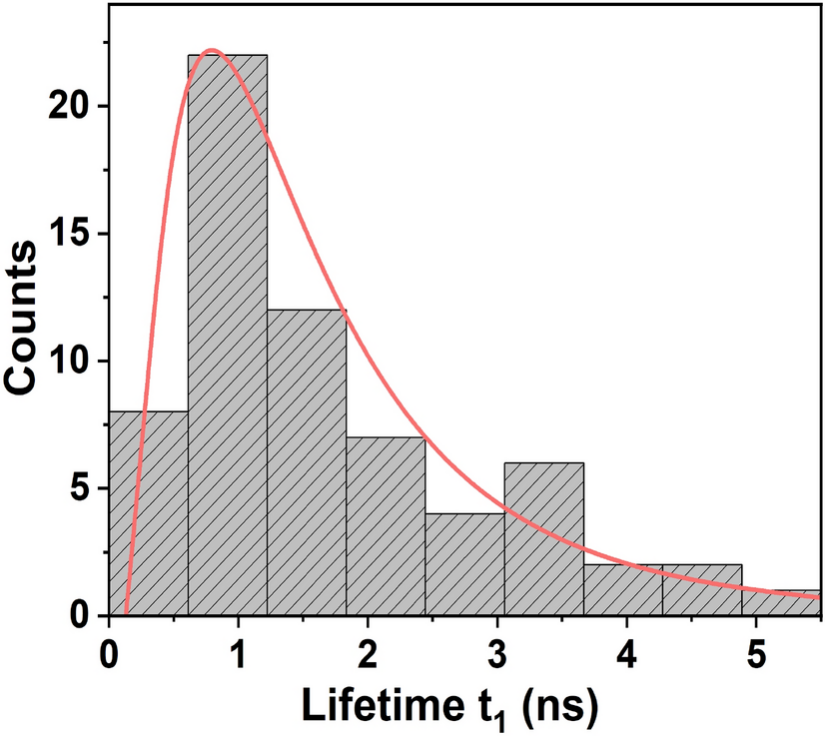
Distribution of lifetimes ($t_1$) from CdSe QDs at 650 nm obtained from $g^{(2)}(\tau )$. Only the correlation data with $g^{(2)}(0) < 0.5$, representing single-photon emission, is considered here. This distribution is fitted (red curve) with the function given in Eq. (7).

The samples used to study single-photon emission in this work are thin films containing CdSe QDs in PMMA prepared via the same procedure. In Fig. 6, we plot the size distribution of the particles in the film, determined from scanning electron microscope image (Supplementary Information (Fig. S1)). The corresponding distribution of single-photon emission lifetimes $t_1$ obtained from the second-order correlation function is presented in Fig. 7.

Theoretically, the rate of spontaneous emission of photons, $\Gamma _{21}$, for the transition between the ground (initial) state $|0 \rangle$ and the excited (final) state $|j \rangle$ is related to the dipole moment matrix elements^41^:

3 $$\begin{aligned} \Gamma _{21} = {\frac{ \omega _j ^3}{{3 \pi \varepsilon _0 \hbar c^3}}| \langle 0| {\varvec{{D}}}|j \rangle | ^2} \end{aligned}$$

If **r** is the displacement vector pointing from the negative charge (electron) to the positive charge (hole) and *e* is the unit charge, then ${\varvec{{D}}} = e \cdot {\varvec{{r}}}$ is the dipole moment that characterizes the strength of interaction between the electron and the surrounding medium. $\langle 0| {\varvec{{D}}}|j \rangle$ refers to the transition dipole moment between the states $| i \rangle$ and $|j \rangle$.

The above expression is valid for the spontaneous emission rate of photons from quantum dots in vacuum. For quantum dots embedded in a matrix, as is the case here, the spontaneous emission rate depends on the dielectric constant $\varepsilon$ of the medium in which it is immersed, and is given by^42,43^

4 $$\begin{aligned} \Gamma _{21}^{\prime } = n \Gamma _{21} = \frac{9 \varepsilon ^{5/2}}{(2 \varepsilon + \varepsilon _{QD})^2} \Gamma _{21} \end{aligned}$$

where $\varepsilon _{QD}$ is the dielectric constant of the QD. Thus the spontaneous emission rate can be increased by immersing the QDs in a medium of higher dielectric constant. In our work, CdSe QDs are embedded in PMMA, a medium having dielectric constant of 2.2^44^, resulting in higher emission rates than reported earlier^45,46^.

According to Eq. (3), $\Gamma _{ij}$ depends quadratically on the dipole moment, and hence the spontaneous emission lifetime of a particular transition is inversely proportional to the size *r* of the particle. The radiative lifetime $t_1 = k/r^2$ where *k* is a constant that depends on the emission frequency,

5 $$\begin{aligned} k = \frac{3 \pi \varepsilon _0 \hbar c^3}{e^2 \omega ^3} \frac{(2 \varepsilon + \varepsilon _{QD})^2}{9 \varepsilon ^{5/2}} \end{aligned}$$

As the dielectric constant of CdSe QDs does not vary significantly over the size range studied^47^, we may compare the lifetime distribution to the particle-size distribution. To this end, we look at the origin of size distribution in the synthesis of CdSe QDs^19^. After rapid injection of the selenium precursor into the cadmium precursor, CdSe nuclei are formed and are homogeneously distributed throughout the solution. These sites have the affinity to attract further molecules due to a greater surface-to-volume ratio and grow rapidly to form QDs. Larger clusters above the critical size ($\approx 2$ nm) attract more molecules, while smaller clusters redissolve. Above the critical size, the smaller particles tend to grow much faster due to a large surface-to-volume ratio (and hence, a higher net charge on the surface). The further growth of larger particles slows down because of insufficient thermal energy to attract further molecules. Hence, the particle-size distribution is skewed towards the smaller particles and a log-normal distribution is favored^48^. If *r* is particle-diameter obtained randomly from SEM image, the probability that *r* takes a range of possible outcomes *R* (such that *r* $\in$ *R*) has a log-normal probability density function given by

6 $$\begin{aligned} P_r(R) = \frac{1}{R \sigma \sqrt{2 \pi }} exp \Biggl \{- \frac{(\ln (R)- \mu )^2}{2 \sigma ^2} \Biggr \} \end{aligned}$$

with median $\mu$ and $\sigma$ is related to the standard deviation of the logarithmic values of the random sizes *r*. The solid red line in Fig. 6, is a log-normal fit to the size distribution with R-squared value of 0.91.

Since the radiative lifetime $t_1 = k/r^2$, the lifetime distribution has a probability density function given by

7 $$\begin{aligned} P_{t_1}(T_1) = \frac{1}{2T_1 \sigma ' \sqrt{2 \pi }} exp \Biggl \{- \frac{(B - \ln (T_1))^2}{8 \sigma '^2} \Biggr \} \end{aligned}$$

where $B=\ln (k) - 2\mu$ is the median of the logarithmic values of random lifetimes $t_1$. In Fig. 7, the lifetime distribution is shown along with a fit to the log-normal distribution. The R-squared value of the fit is 0.99, indicating a good agreement with Eq. (7). An exciton in a larger QD exhibits a larger optical dipole moment when compared to a smaller QD. A consequence of having a large optical dipole moment is the rapid decay of excitons^33^, leading to a shorter lifetime. The lifetime distribution in Fig. 7 may be compared with the shaded region in size distribution in Fig. 6. The size distribution peaks at 10.5 nm, while the lifetime distribution has its peak at 0.8 ns. From a comparison of these two distributions, we may infer that lifetimes lower than that of 0.8 ns are exhibited by particles having a size larger than 10.5 nm.

The second-order correlation function is calculated for 83 particles, of which 64 particles resulted in $g^{(2)}(0) < 0.5$, implying a highly reproducible single-photon emission from the CdSe QDs in the film. The value of $g^{(2)}(0)$ ranges from 0.03 to 0.22. Those particles that lead to $g^{(2)}(0) > 0.5$ might have a size larger than 11 nm^33^ which is approximately the exciton Bohr diameter of CdSe. The particles above this size correspond to a weaker exciton binding energy, with a higher probability of multiple-photon emission. Strong confinement of carriers in CdSe QDs would occur below the exciton Bohr diameter, beyond which the probability of multiple-photon generation is high. Hence, a distribution of particle size up to 11 nm is shown in Fig. 6 for comparison with the lifetime distribution. It is noticed that the lifetime is negatively correlated with the size of the particle, which is in agreement with the relation described in Eq. (3).

The strength of the monotonic relationship between the particle-size and the radiative lifetime is assessed by Spearman’s rank correlation coefficient $\rho$, which takes the values $(-1 \le \rho \le +1)$, where +1 corresponds to a perfect positive correlation, and − 1 to a perfect negative correlation. The value zero indicates the absence of any correlation. To calculate $\rho$, the curve fitting functions for the size (*r*) distribution and the lifetime (*t*) distributions are discretized into 100 data points. The discrete data points for both size and lifetime distributions exhibit one-to-one correspondence through the relation $t_1 = k/r^2$. If $d_i$ is the difference between the ranks of the $i^{th}$ pair of values of *r* and $t_1$, and *n* is the largest rank, then the Spearman’s rank correlation coefficient is calculated using

8 $$\begin{aligned} \rho = 1 - \frac{6}{n(n^2 -1)}\sum d_i^2 \end{aligned}$$

**Fig. 8:**
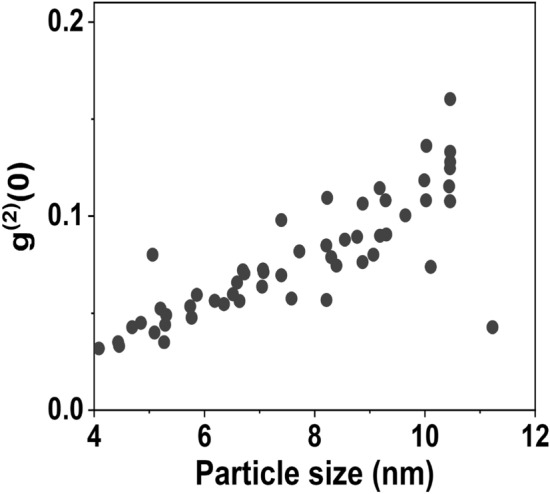
Plot of $g^{(2)}(0)$ obtained using HBT experiment versus size obtained using $r = \sqrt{k/t_1}$. Single photons are emitted from the particles in the confinement regime, hence $g^{(2)}(0) < 0$. The probability of multiple-photon emission increases with increase in the particle-size and it results in increase in $g^{(2)} (0)$.

For the present data, $\rho$ is estimated to be $-0.594$, which indicates that the particle-size and spontaneous emission lifetime are negatively correlated. The good correlation between the size distribution and the lifetime distribution may prompt us to hazard an estimate of the constant *k* in the relation $t_1 = k/r^2$. Within the error limits of the bin width, We can safely assume that the peak value seen at 0.8 ns in the lifetime distribution corresponds to particles of size 10.5 nm, where the maximum number of particles are observed in the size distribution. This tells us that the value of *k* is equal to $9 \pm 3 \times 10^{-26}$. This analysis shows that the sizes of the quantum dots studied by us vary from $14 \pm 2$ nm (for $t_1 = 0.4$ ns) to $4.1 \pm 0.6$ nm (for $t_1 = 5.2$ ns).

Estimating the size of nanoparticles embedded in dielectric films is important for various experiments and applications. However, optical imaging technique does not have the resolution to determine the size of nanoparticles in the size range below the diffraction limit. Estimating the size of these particles is a major challenge due to the special sample preparation techniques needed for electron microscopes. A monotonic relationship between the particle size distributions of identically prepared nanoparticles and their optical absorption properties has been established elsewhere^49^. Due to the strong correlation between particle-size and spontaneous emission lifetime, the method discussed above could be a promising approach to estimate the size of nanoparticles embedded in a thin film using optical methods. In Fig. 8, we plot $g^{(2)} (0)$ for the particles studied against the estimated size. The plot shows that $g^{(2)} (0)$ increases with size and also that there is a larger fluctuation in the value of $g^{(2)} (0)$ for larger particle size. This behavior further demonstrates the fact that the probability of multiple-photon emission increases with increasing particle-size, which is found elsewhere^50^.

## Summary and conclusion

In this work, we study the single-photon emission characteristics of colloidal CdSe QDs synthesized using a green synthesis protocol. The spontaneous emission lifetime of these QDs is estimated by analyzing the second-order correlation function $g^{(2)}(\tau )$ measured using an HBT setup attached to an in-house constructed confocal microscope, whose values are verified with the time-resolved technique. An order of magnitude reduction in the lifetime is achieved by embedding CdSe QDs in a medium of higher dielectric constant. The value of $g^{(2)}(0)$ ranging from 0.03 to 0.22 at room temperature for 64 trials out of 83 trials indicates high-quality single-photon emission. By comparing the CdSe QD size distribution with the lifetime distribution, we could estimate the size of the CdSe QDs from their single-photon emission lifetime. From this analysis, we could determine the value of $g^{(2)}(0)$ as a function of QD size, and it is observed that $g^{(2)}(0)$ increases with QD size. A larger spread is observed in the $g^{(2)}(0)$ values as the size of the QD increases. This observation is supported by the fact that there is a larger probability of multiple-photon emission as the QD size increases.

## Supplementary Information

Supplementary Information.

## Data Availability

The datasets used and analyzed during the current study are available from the corresponding author on reasonable request.

## References

[1] Neeleshwar, S. et al. Size-dependent properties of CdSe quantum dots. *Phys. Rev. B* **71**, 201307 (2005).

[2] Hao, E. et al. Synthesis and optical properties of CdSe and CdSe/CdS nanoparticles. *Chem. Mater.* **11**, 3096–3102 (1999).

[3] Yang, Y. et al. High-efficiency light-emitting devices based on quantum dots with tailored nanostructures. *Nat. Photonics* **9**, 259–266 (2015).

[4] Lai, R., Pu, C. & Peng, X. On-surface reactions in the growth of high-quality CdSe nanocrystals in nonpolar solutions. *J. Am. Chem. Soc.* **140**, 9174–9183 (2018). 29956924 10.1021/jacs.8b04743

[5] Han, Z., Qiu, F., Eisenberg, R., Holland, P. L. & Krauss, T. D. Robust photogeneration of h2 in water using semiconductor nanocrystals and a nickel catalyst. *Science* **338**, 1321–1324 (2012). 23138979 10.1126/science.1227775

[6] Brus, L. Electronic wave functions in semiconductor clusters: Experiment and theory. *J. Phys. Chem.* **90**, 2555–2560 (1986).

[7] Meulenberg, R. W. et al. Determination of the exciton binding energy in CdSe quantum dots. *ACS Nano* **3**, 325–330 (2009). 19236067 10.1021/nn8006916

[8] Efros, A. L. & Efros, A. L. Interband absorption of light in a semiconductor sphere. *Sov. Phys. Semicond.* **16**, 772–775 (1982).

[9] Morrison, C. L. et al. A bright source of telecom single photons based on quantum frequency conversion. *Appl. Phys. Lett.* **118**, 174003 (2021).

[10] Von Helversen, M. et al. Quantum metrology of solid-state single-photon sources using photon-number-resolving detectors. *New J. Phys.* **21**, 035007 (2019).

[11] Braig, C., Zarda, P., Kurtsiefer, C. & Weinfurter, H. Experimental demonstration of complementarity with single photons. *Appl. Phys. B* **76**, 113–116 (2003).

[12] Tomm, N. et al. A bright and fast source of coherent single photons. *Nat. Nanotechnol.* **16**, 399–403 (2021). 33510454 10.1038/s41565-020-00831-x

[13] Proppe, A. H. et al. Highly stable and pure single-photon emission with 250 ps optical coherence times in InP colloidal quantum dots. *Nat. Nanotechnol.* **18**, 993–999 (2023). 37386140 10.1038/s41565-023-01432-0

[14] Hoang, T. B., Akselrod, G. M. & Mikkelsen, M. H. Ultrafast room-temperature single photon emission from quantum dots coupled to plasmonic nanocavities. *Nano Lett.* **16**, 270–275 (2016). 26606001 10.1021/acs.nanolett.5b03724

[15] Naiki, H., Masuo, S., Machida, S. & Itaya, A. Single-photon emission behavior of isolated CdSe/ZnS quantum dots interacting with the localized surface plasmon resonance of silver nanoparticles. *J. Phys. Chem. C* **115**, 23299–23304 (2011).

[16] Zhou, X., Tamargo, M., Guo, S. & Chen, Y. Optical properties of CdSe quantum dots grown on ZnSe and ZnBeSe by molecular beam epitaxy. *J. Electron. Mater.* **32**, 733–736 (2003).

[17] Guo, L., Zhang, S., Feng, W., Hu, G. & Li, W. A first-principles study on the structural, elastic, electronic, optical, lattice dynamical, and thermodynamic properties of zinc-blende cdx (x= s, se, and te). *J. Alloy. Compd.* **579**, 583–593 (2013).

[18] Murray, C., Norris, D. J. & Bawendi, M. G. Synthesis and characterization of nearly monodisperse CdE (E= sulfur, selenium, tellurium) semiconductor nanocrystallites. *J. Am. Chem. Soc.* **115**, 8706–8715 (1993).

[19] Van Embden, J., Chesman, A. S. & Jasieniak, J. J. The heat-up synthesis of colloidal nanocrystals. *Chem. Mater.* **27**, 2246–2285 (2015).

[20] Yard, P. et al. On-chip quantum information processing with distinguishable photons. *Phys. Rev. Lett.* **132**, 150602 (2024). 38682995 10.1103/PhysRevLett.132.150602

[21] Novotny, L. & Hecht, B. *Principles of nano-optics* (Cambridge University Press, Cambridge, 2012).

[22] Boles, M. A., Ling, D., Hyeon, T. & Talapin, D. V. The surface science of nanocrystals. *Nat. Mater.* **15**, 141–153 (2016). 26796733 10.1038/nmat4526

[23] Fritzinger, B., Capek, R. K., Lambert, K., Martins, J. C. & Hens, Z. Utilizing self-exchange to address the binding of carboxylic acid ligands to CdSe quantum dots. *J. Am. Chem. Soc.* **132**, 10195–10201 (2010). 20608680 10.1021/ja104351q

[24] dos Santos, J. A. L. et al. 3-mercaptopropionic, 4-mercaptobenzoic, and oleic acid-capped CdSe quantum dots: Interparticle distance, anchoring groups, and surface passivation. *J. Nanomater.* **2019**, 2796746 (2019).

[25] Yuan, G., Gómez, D. E., Kirkwood, N., Boldt, K. & Mulvaney, P. Two mechanisms determine quantum dot blinking. *ACS Nano* **12**, 3397–3405 (2018). 29579376 10.1021/acsnano.7b09052

[26] Efros, A. L. & Nesbitt, D. J. Origin and control of blinking in quantum dots. *Nat. Nanotechnol.* **11**, 661–671 (2016). 27485584 10.1038/nnano.2016.140

[27] Yang, C. *et al.* Mechanisms and suppression of quantum dot blinking. *Laser Photonics Rev*. 2402269. 10.1002/lpor.202402269 (2025).

[28] Hohng, S. & Ha, T. Near-complete suppression of quantum dot blinking in ambient conditions. *J. Am. Chem. Soc.* **126**, 1324–1325 (2004). 14759174 10.1021/ja039686w

[29] Chen, Y. et al. “giant’’ multishell CdSe nanocrystal quantum dots with suppressed blinking. *J. Am. Chem. Soc.* **130**, 5026–5027 (2008). 18355011 10.1021/ja711379k

[30] Shi, J. et al. All-optical fluorescence blinking control in quantum dots with ultrafast mid-infrared pulses. *Nat. Nanotechnol.* **16**, 1355–1361 (2021). 34811550 10.1038/s41565-021-01016-w

[31] Ji, Z. & Song, Z. Exciton radiative lifetime in CdSe quantum dots. *J. Semicond.* **44**, 032702 (2023).

[32] Singhal, P. & Pulhani, V. Effect of ligand concentration, dilution, and excitation wavelength on the emission properties of CdSe/CdS core shell quantum dots and their implication on detection of uranium. *ChemistrySelect* **4**, 4528–4537 (2019).

[33] Gong, K., Zeng, Y. & Kelley, D. F. Extinction coefficients, oscillator strengths, and radiative lifetimes of CdSe, CdTe, and CdTe/CdSe nanocrystals. *J. Phys. Chem. C* **117**, 20268–20279 (2013).

[34] Engineer, S. et al. Certifying high-dimensional quantum channels. arXiv preprint arXiv:2408.15880 (2024).

[35] Sapra, S., Rogach, A. L. & Feldmann, J. Phosphine-free synthesis of monodisperse CdSe nanocrystals in olive oil. *J. Mater. Chem.* **16**, 3391–3395 (2006).

[36] Shakhi, P., Bijeesh, M., Varier, G. K. & Nandakumar, P. An in-house constructed dual channel confocal fluorescence microscope for biomolecular imaging. *OSA Continuum* **4**, 2177–2192 (2021).

[37] Nirmal, M. et al. Fluorescence intermittency in single cadmium selenide nanocrystals. *Nature* **383**, 802–804 (1996).

[38] Zhao, S. et al. Single photon emission from graphene quantum dots at room temperature. *Nat. Commun.* **9**, 3470 (2018). 30150689 10.1038/s41467-018-05888-w PMC6110725

[39] Goldzak, T., McIsaac, A. R. & Van Voorhis, T. Colloidal CdSe nanocrystals are inherently defective. *Nat. Commun.* **12**, 890 (2021). 33563985 10.1038/s41467-021-21153-z PMC7873310

[40] Nair, G., Zhao, J. & Bawendi, M. G. Biexciton quantum yield of single semiconductor nanocrystals from photon statistics. *Nano Lett.* **11**, 1136–1140 (2011). 21288042 10.1021/nl104054t PMC3278281

[41] Van Driel, A. et al. Frequency-dependent spontaneous emission rate from CdSe and CdTe nanocrystals: Influence of dark states. *Phys. Rev. Lett.* **95**, 236804 (2005). 16384329 10.1103/PhysRevLett.95.236804

[42] Thränhardt, A., Ell, C., Khitrova, G. & Gibbs, H. Relation between dipole moment and radiative lifetime in interface fluctuation quantum dots. *Phys. Rev. B* **65**, 035327 (2002).

[43] Yablonovitch, E., Gmitter, T. & Bhat, R. Inhibited and enhanced spontaneous emission from optically thin AlGaAs/GaAs double heterostructures. *Phys. Rev. Lett.* **61**, 2546 (1988). 10039153 10.1103/PhysRevLett.61.2546

[44] Polyanskiy, M. N. Refractiveindex. info database of optical constants.. *Sci. Data* **11**, 94 (2024). 38238330 10.1038/s41597-023-02898-2 PMC10796781

[45] Fedin, I. et al. Enhanced emission from bright excitons in asymmetrically strained colloidal CdSe/Cd_x_Zn_1-x_Se quantum dots. *ACS Nano* **15**, 14444–14452 (2021). 34473467 10.1021/acsnano.1c03864

[46] Morozov, S. et al. Purifying single photon emission from giant shell CdSe/CdS quantum dots at room temperature. *Nanoscale* **15**, 1645–1651 (2023). 36597874 10.1039/d2nr04744f

[47] Casas Espínola, J. & Hernández Contreras, X. Effect of dielectric constant on emission of CdSe quantum dots. *J. Mater. Sci.: Mater. Electron.* **28**, 7132–7138 (2017).

[48] Micic, O. I., Curtis, C. J., Jones, K. M., Sprague, J. R. & Nozik, A. J. Synthesis and characterization of InP quantum dots. *J. Phys. Chem.* **98**, 4966–4969 (1994).

[49] Arunkarthick, S., Bijeesh, M., Varier, G. K., Kowshik, M. & Nandakumar, P. Laser scanning photothermal microscopy: Fast detection and imaging of gold nanoparticles. *J. Microsc.* **256**, 111–116 (2014). 25179372 10.1111/jmi.12164

[50] Igarashi, H., Yamauchi, M. & Masuo, S. Correlation between single-photon emission and size of cesium lead bromide perovskite nanocrystals. *J. Phys. Chem. Lett.* **14**, 2441–2447 (2023). 36862129 10.1021/acs.jpclett.3c00059

