# Supplementary material for: Influence of particle size and dielectric environment on radiative lifetimes of colloidal cadmium selenide single photon emitters
Source: Sci Rep. 2025 Apr 26;15:14639. doi: 10.1038/s41598-025-99148-9 (PMC12033363; doi:10.1038/s41598-025-99148-9)
Supplement: Supplementary file 1 — Supplementary Information. [file 41598_2025_99148_MOESM1_ESM.pdf]

Influence of Particle Size and Dielectric Environment on Radiative Lifetimes of Colloidal Cadmium Selenide Single Photon Emitters

V. Manojkumar[1], Geetha K. Varier[1], Radhika Vathsan[1], and P. Nandakumar*[1]

*nandan@goa.bits-pilani.ac.in
[1]Department of Physics, Birla Institute of Technology and Science, Pilani, K K Birla Goa Campus, Zuarinagar, Sancoale, Goa 403726, India

Supplementary Information 1: Scanning Electron Microscope Image

The thin film of colloidal CdSe QDs is imaged as shown in Figure S1 using scanning electron microscope (1 nm resolution, FEI Quanta FEG 250) for size characterisation. A closer observation of individual spots in the image reveals largely monodisperse quantum dots even though a few agglomerated clusters are also present. The average size of 10 ± 1 nm from 200 monodisperse CdSe QDs with a polydispersity index of 0.11, indicates a narrow size distribution obtained from heat-up synthesis.

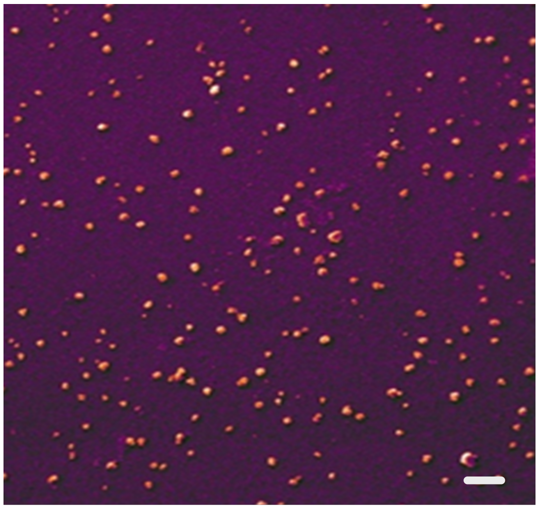

Figure S1. SEM image of colloidal CdSe QDs. Bright spots are the individual particles. The scale bar is 50 nm and the average size of particles is 10 ± 1 nm.

Supplementary Information 2: Absorption and Photoluminescence Spectroscopy

Absorption spectrum of the colloidal CdSe QDs is studied using UV-vis spectrometer (V-750, Jasco Inc.) (Figure S2 (a)). A bandgap energy of 1.95 eV is obtained from the corresponding Tauc plot (inset of Figure S2 (a)), which is greater than that of the bulk cadmium selenide (1.74 eV). The increase in the bandgap is due to the quantum confinement effects.

Figure S2 (b) shows the photoluminescence spectrum of CdSe QDs obtained using Horiba LabRAM HR Evolution spectrometer. A solution containing CdSe QDs is excited with a laser beam having a wavelength of 532 nm. The emission peak observed at 650 nm shows red-shift with increase in temperature and is attributed to the band-edge emission. A weak shoulder peak at around 580 nm which gets suppressed with increase in temperature could be due to higher excited states of QDs or ligands[1]. The defect state emissions in this CdSe QDs solution are very weak and not seen in the photoluminescence spectrum. The photoluminescence emission is a strong function of the size of CdSe QDs. Due to the presence of QDs of different sizes in the solution, and since each QD emits at slightly different frequencies, the resulting spectrum is inhomogeneously broadened.

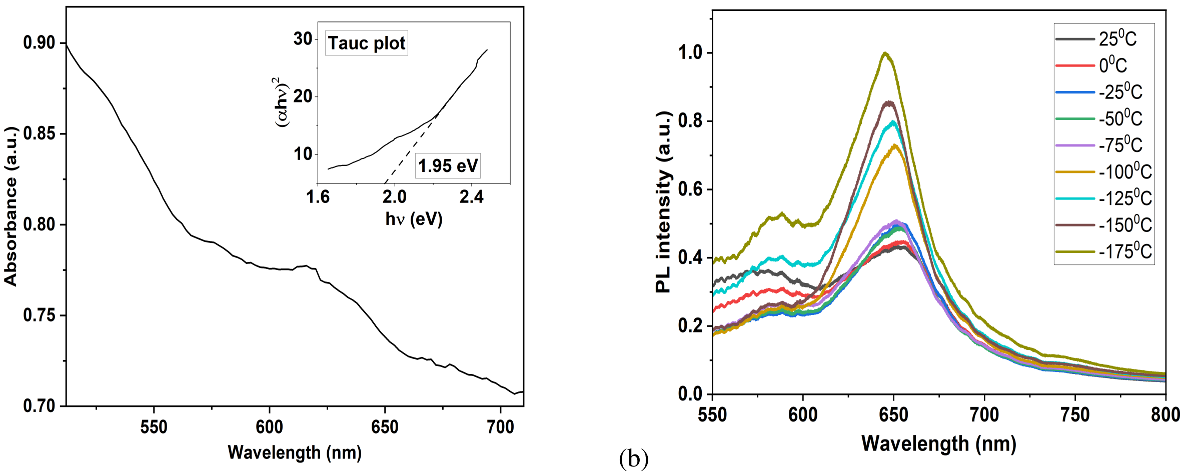

(a) (b)

Figure S2. **(a)** Absorption spectrum of CdSe quantum dots with the corresponding Tauc plot shown in the inset. **(b)** Photoluminescence spectrum of CdSe QDs excited using 532 nm light.

References

1. Çadırcı, M. Temperature-dependent photoluminescence of cdse/cdte quasi-type-ii quantum dots. *J. Lumin.* **228**, 117551 (2020).

Supplementary Data 3: Second-order correlation function for a few trials

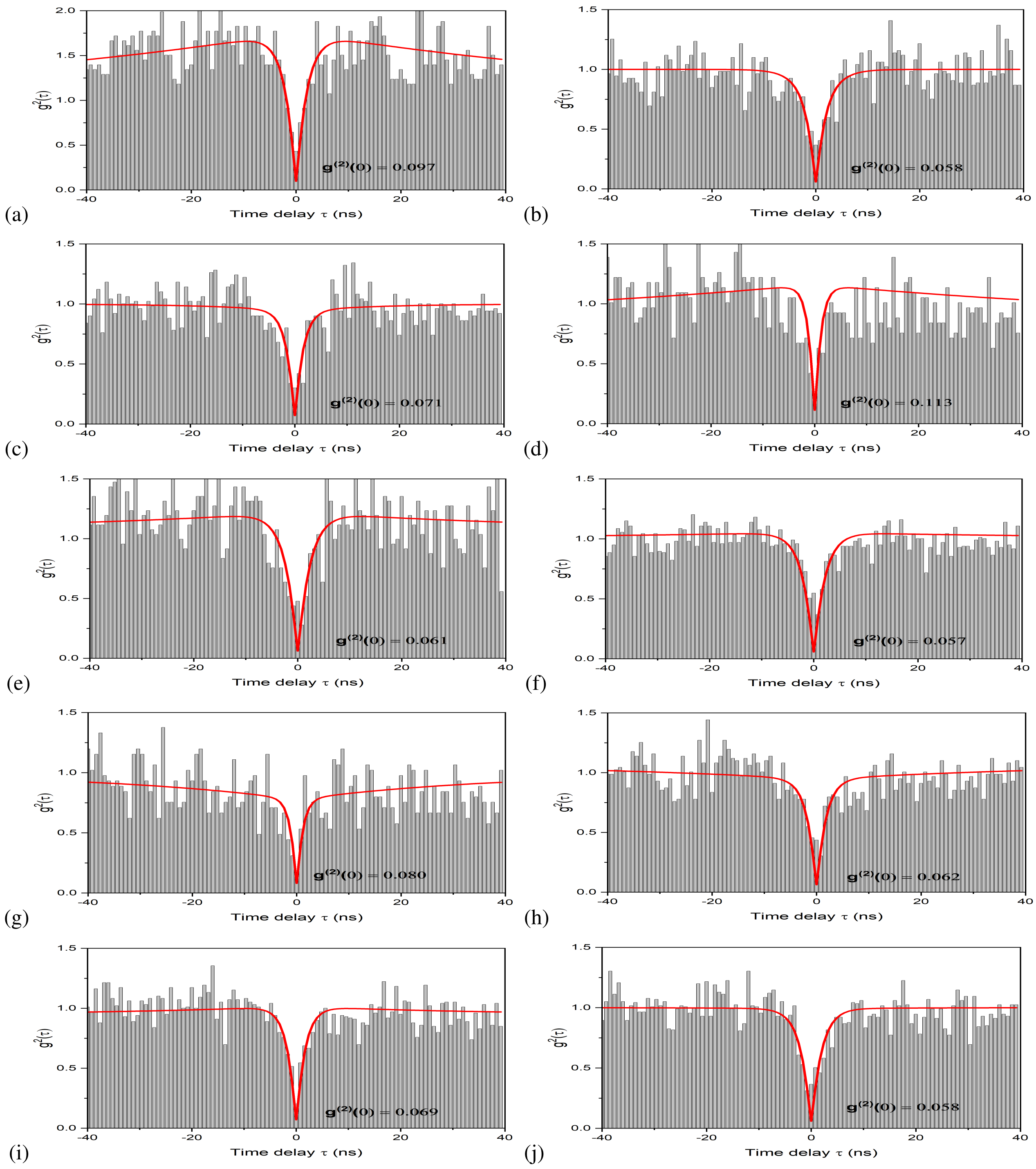

Figure S3. Second order correlation function $g^2(\tau)$ as a function of time-delay τ for a few colloidal cadmium selenide quantum dots.
